# Anti-icing strategies of plant surfaces: the ice formation on leaves visualized by Cryo-SEM experiments

**DOI:** 10.1007/s00114-022-01789-7

**Published:** 2022-04-04

**Authors:** Stanislav N. Gorb, Elena V. Gorb

**Affiliations:** Department of Functional Morphology and Biomechanics, Zoological Institute, Kiel University, Am Botanischen Garten 9, 24118 Kiel, Germany

**Keywords:** Microstructure, Nanostructure, Trichomes, 3D wax projections, Wettability, Biomimetics

## Abstract

This paper presents an experimental study on surface icing on leaves in six plant species having different surface micromorphology and wettability properties. Contrary to previous studies on ice crystallization, which have been mainly performed by using infrared video thermography, we applied a Cryo-SEM approach allowing not only characterization of plant surfaces in their native conditions but also visualization of ice crystal formation on the native plant surfaces at the micro- and nanoscales. The Cryo-SEM was also used as an experimental device to freeze water vapor, thaw ice crystals, and freeze fluid water on the plant surface again. The experiments clearly demonstrate that trichome coverage (especially with several distinct layers) and 3D wax projections can be recognized as anti-icing strategies of plants. Trichomes can prevent and delay ice formation by being nucleation points for the formation of ice from vapor and protect the plant surface from overcooling, when fluid water freezes in contact with the leaf surface. The study shows for the first time two important effects that might reduce plant cell freezing rate: the presence of air pockets between wax projections that protect from direct contact between ice crystals and the plant cuticle and elimination of fluid water after thawing and preventing further re-freezing on the surface. The detailed knowledge obtained here is not only important for plant ecology, evolution, and plant protection but also for looking for potential biomimetic strategies that reduce/avoid icing of cultural plants and artificial technical surfaces.

**Graphical Abstract:**

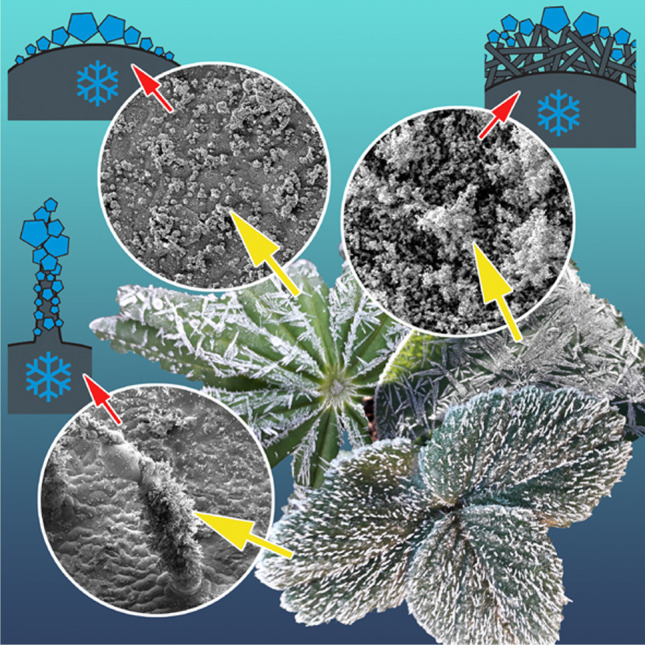

## Introduction

**Fig. 1:**
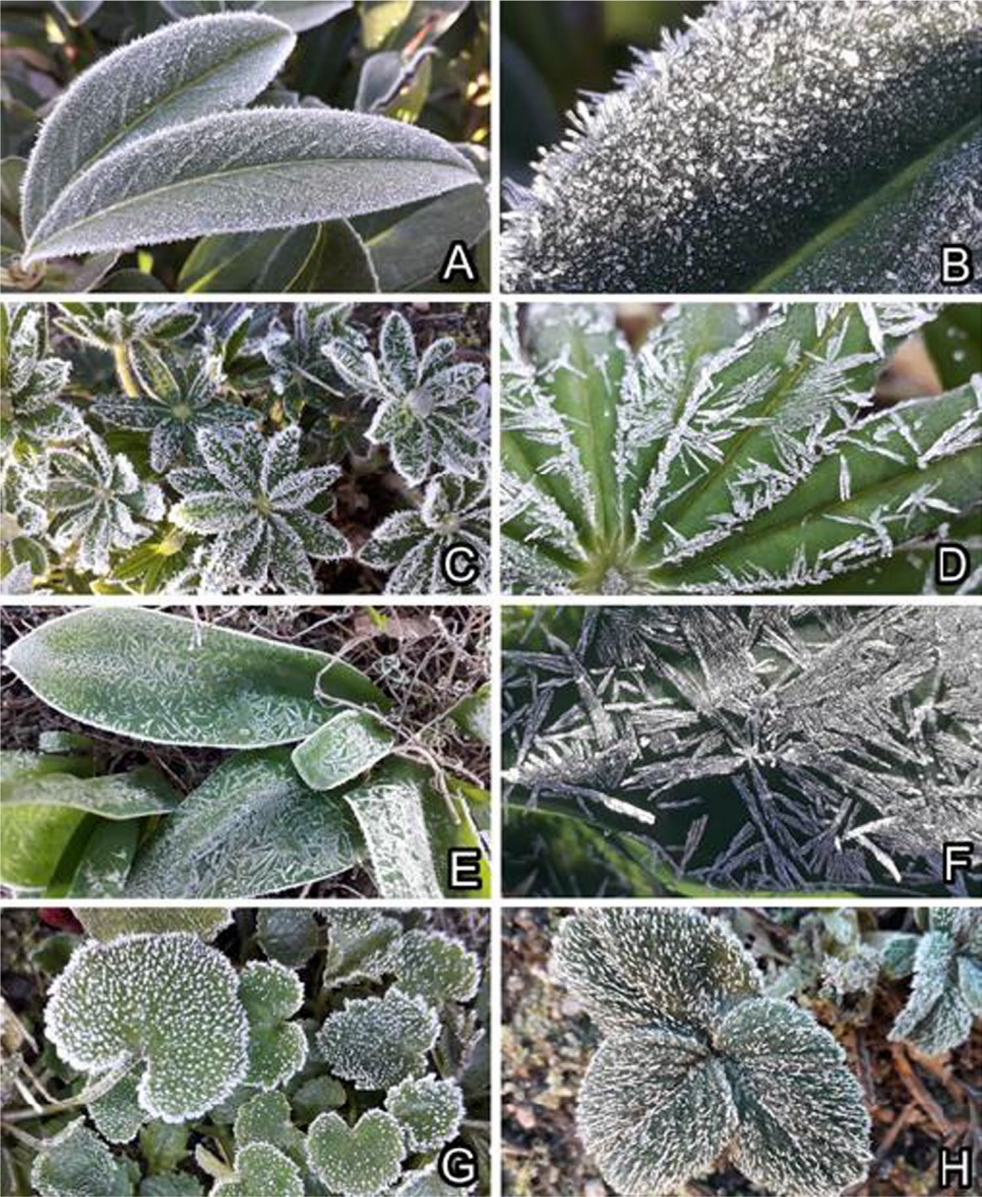
Natural occurrence of ice on plant leaf surfaces in early spring: *Prunus laurocerasus* (**A**, **B**), *Lupinus polyphyllus* (**C**, **D**), *Tulipa gesneriana* (**E**, **F**), *Campanula portenschlagiana* (**G**), and *Fragaria ananassa* (**H**)

Vegetative and generative organs of many herbaceous plant species of temperate zones are often exposed to temperatures below zero and can be easily iced on their surfaces (Fig. 1). In order to prevent the freezing of their cells at the outside temperatures below zero, plants evolved different mechanisms: the use of antifreezes (substances that shift the freezing point of water to lower temperatures) and the presence of ice nucleators or anti-nucleators (Arora and Rowland 2011; Gusta and Wisniewski 2013; Bredow et al. 2016; Bredow and Walker 2017). The preventing of ice formation within the cells is the most crucial goal for plants to survive freezing events. The formation of extracellular ice in intercellular spaces is one of the widely studied adaptations against plant cell freezing (Wisniewski et al. 1997, 2002a, b, 2014; Wisniewski and Fuller 1999; Gusta and Wisniewski 2013; Schott et al. 2017). The extracellular ice formation was previously examined in different organs and plants including herbaceous plant species (McCully et al. 2000) and coniferous trees (Ball et al. 2004; Roden et al. 2009).

However, ice can also be easily formed on the outer surfaces of plants and this ice can form quickly and in large amounts. The epicuticular wax layer, cuticle, and cell walls of epidermal cells can prevent the ice crystal’s nucleation from moving inside cells and damaging them, but some plant cuticles are rather thin and vulnerable toward potential mechanical damage from ice crystals. Moreover, plant surfaces are rather different in their physico-chemical properties (hydrophilic, hydrophobic, and superhydrophobic), which certainly might have an effect on ice nucleation on the plant surface. Especially less hydrophobic surfaces, having strong contact with water when plants are initially wet, may generate heavy crusts of ice when environmental temperatures go below zero presenting a potential damage source for the plant surface. It has also been previously reported that plant surfaces, made hydrophobic by artificial particles, limited the spreading of ice crystal formation and delayed plant freezing (Fuller et al. 2003; Kuprian et al. 2016). On the other hand, the application of acrylic polymer films led to the same amount or even more damage, when compared to control plants. Subsequent examination of the freezing of leaves revealed that the hydrophobic particle film delayed the entry of ice from a frozen water droplet. It has been concluded that the hydrophobic particle film shows considerable promise as a frost protection agent that might be applied to plants prior to a freezing event (Fuller et al. 2003).

Plant surfaces show a huge diversity in their micro- and nanostructure and in their physico-chemical properties: some of them are rather smooth and have a film-like wax coverage; the other ones are more or less densely covered with trichomes or/and 3D epicuticular waxes (Gorb and Gorb 2002, 2017). That is why it is plausible to assume that many plants have natural adaptation of their surfaces against icing. However, our knowledge about ice formation and the related behavior on plant surfaces having different physico-chemical properties is rather limited. In the present study, we aimed at visualization of icing on plant surfaces having different micro- and nanostructures. We selected leaves of six plant species with rather smooth surfaces (2) and those covered with trichomes (2) or epicuticular wax projections (2). In nature, leaves of all these plants, in spite of their different surface properties, may be potentially exposed to freezing temperatures and are rather successful in surviving under these conditions. With this comparative approach, we wanted to understand the formation of ice crystals depending on surface properties and to recognize functional principles in preventing or altering ice damage by different plant surfaces.

Previously, studies on ice crystallization have been performed by using infrared video thermography (Wisniewski et al. 1997; Pearce 2001; Pearce and Fuller 2001; Fuller et al. 2003) and only in a few limited cases by using cryogenic scanning electron microscopy (Cryo-SEM) (Pearce 1988; McCully et al. 2000). A Cryo-SEM approach provides a powerful methodology to not only characterize plant surfaces in their native conditions (Gorb and Gorb 2019; Rebora et al. 2020a, b; Salerno et al. 2018, 2019, 2020) but also to visualize ice crystal formation within or on the native plant surfaces at the micro- and nanoscales (Pearce 2001; Schott et al. 2017). In the present study, Cryo-SEM was used as an experimental device to (1) freeze water vapor, (2) thaw ice crystals, (3) freeze fluid water on the plant surface again, and (4) simultaneously visualize interactions between the ice and the surface.

The knowledge about freezing processes on plant surfaces is not only important for plant ecology, evolution, and plant protection (Fuller et al. 2003) but also for potential biomimetic strategies that reduce/avoid icing of cultural plants/crops and artificial surfaces (see review by Li and Guo (2018)).

## Methods

### Plants

**Fig. 2:**
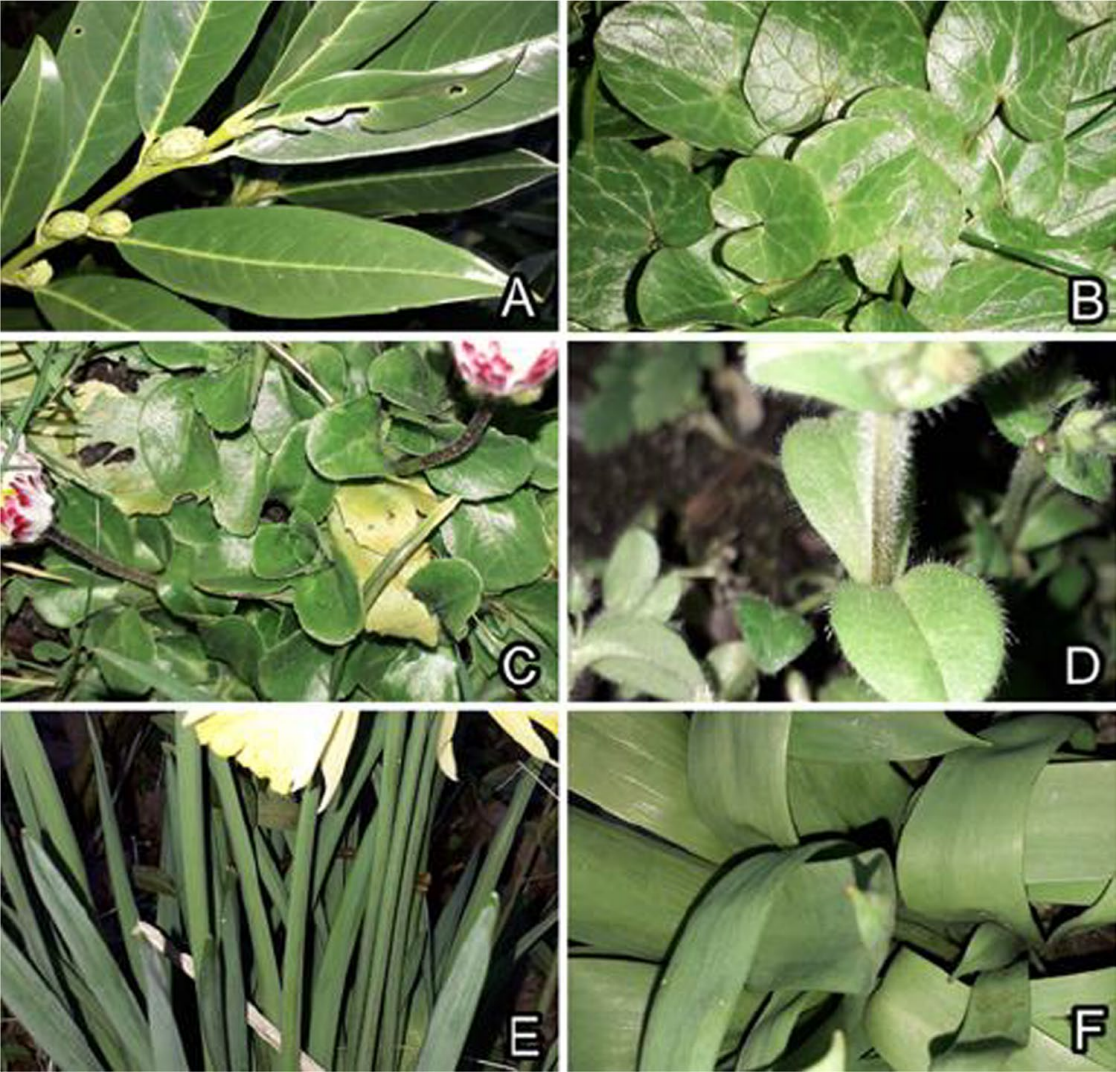
Plants used in the Cryo-SEM experiments: *Prunus laurocerasus* (**A**), *Ficaria verna* (**B**), *Bellis perennis* (**C**), *Cerastium brachypetalum* (**D**), *Narcissus pseudonarcissus* (**E**), and *Tulipa gesneriana* (**F**)*.* Smooth surfaces (**A**, **B**). Surfaces covered with trichomes (**C**, **D**). Surfaces covered with 3D epicuticular waxes (**E**, **F**)

**Fig. 3:**
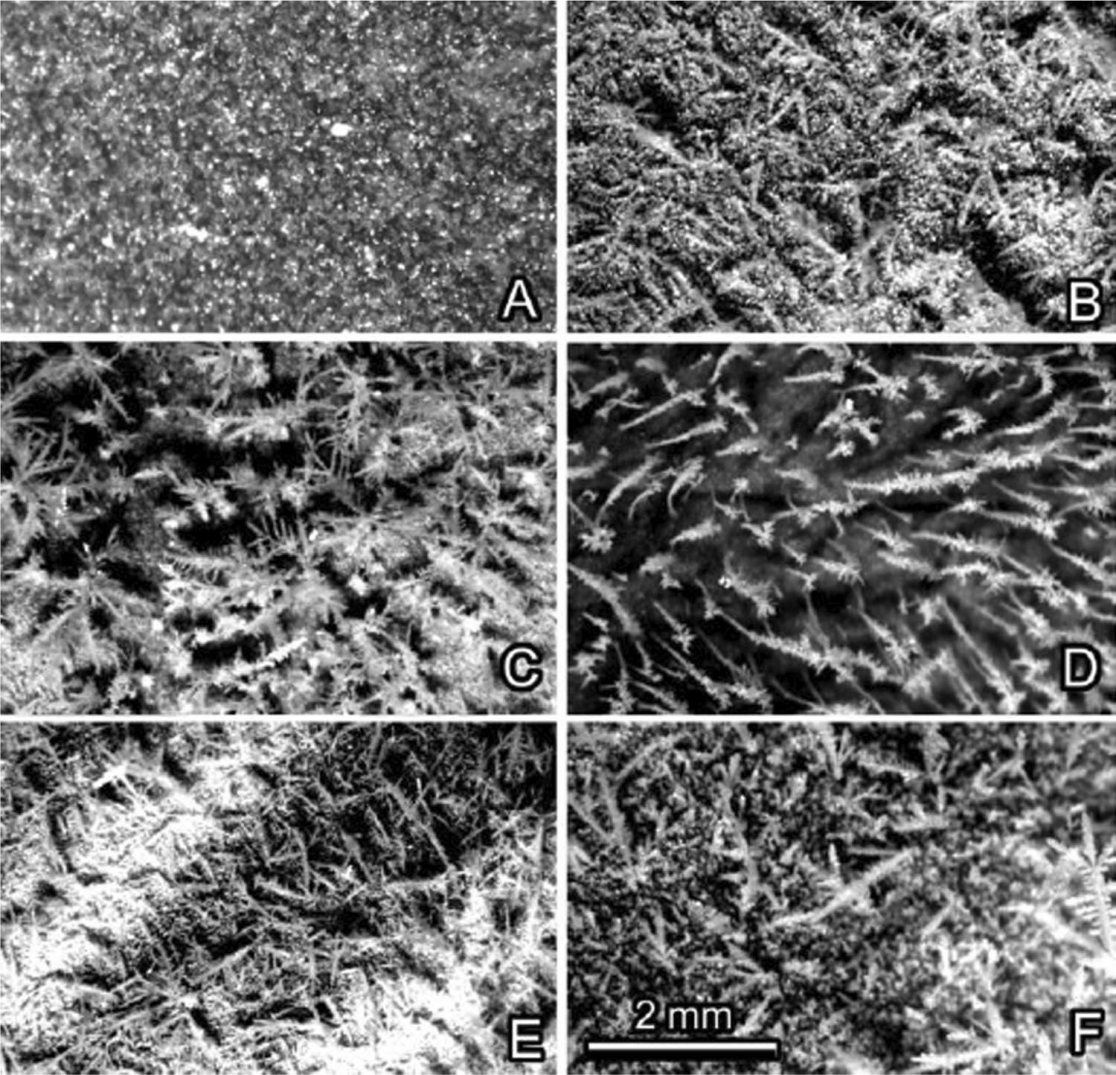
‘Icing’ of experimental plant leaf surfaces (images taken in the binocular microscope): *Prunus laurocerasus* (**A**), *Ficaria verna* (**B**), *Bellis perennis* (**C**), *Cerastium brachypetalum* (**D**), *Tulipa gesneriana* (**E**, **F**)*.* Smooth surfaces (**A**, **B**). Surfaces covered with trichomes (**C**, **D**). Surface covered with 3D epicuticular wax (**E**, **F**)

Natural occurrence of ice on leaf surfaces of plants was studied in early Spring in the Botanical Garden of the University of Kiel (Kiel, Germany). The following species were observed, and icing on their leaves was documented by taking images in nature and at various magnifications under the binocular microscope (Figs. 1, 2, and 3): *Prunus laurocerasus* (Rosaceae), *Fragaria ananassa* (Rosaceae), *Lupinus polyphyllus* (Fabaceae), *Campanula portenschlagiana* (Campanulaceae), *Ficaria verna* (Ranunculaceae), *Bellis perennis* (Asteraceae), *Cerastium brachypetalum* (Caryophyllaceae), *Narcissus pseudonarcissus* (Amaryllidaceae), and *Tulipa gesneriana* (Liliaceae)*.* Leaves of the following species were collected and their upper (adaxial) surfaces were used in the Cryo-SEM experiments (Fig. 2): *P. laurocerasus* and *F. verna* (smooth surfaces), *B. perennis* and *C. brachypetalum* (surfaces covered with trichomes), and *N. pseudonarcissus* and *T. gesneriana* (surfaces covered with 3D epicuticular waxes)*.*

### Cryo-SEM examination of native plant surfaces

Native surfaces of plant leaves were examined using cryo scanning electron microscopy (SEM). Small samples (1 cm × 1 cm) from the middle part of the leaves were cut out from living plants, immediately attached with their lower (abaxial) side to the metal holder using polyvinyl alcohol Tissue-Tek® OCTTM compound (Sakura Finetek Europe, Zoeterwoude, The Netherlands), and frozen in a cryo-stage preparation chamber at − 140 °C (Gatan ALTO 2500 cryo preparation system, Gatan Inc., Abingdon, UK). Frozen samples were sputter-coated with gold–palladium (thickness 6 nm) and studied in the frozen condition in a Cryo-SEM Hitachi S-4800 (Hitachi High-Technologies Corporation, Tokyo, Japan) at 3 kV accelerating voltage and − 120 °C temperature. Description of the trichomes was performed mainly according to Voigt et al. (2007). Types of wax projections were identified according to the classification of plant epicuticular waxes proposed by Barthlott et al. (1998). Morphometrical variables of wax projections were measured from digital images using the image analysis software SigmaScan Pro Version 5.0.0 (SPSS Inc., Chicago, USA) and presented in the text as mean ± SD for *n* = 10 leaves of 10 individual plants for each species.

### Contact angle measurements on native plant surfaces

Contact angles of double-distilled water (density = 1.000 kg m^−3^, surface tension = 72.1 mN m^−1^, dispersion component = 19.9 mN m^−1^, polar component = 52.2 mN m^−1^; Busscher et al. 1984) were measured in the middle regions on the upper leaf surface in six plant species studied. We applied a high-speed optical contact angle measuring device OCAH 200 (DataPhysics Instruments GmbH, Filderstadt, Germany) and used sessile (for smooth and hairy surfaces) or sessile needle-in (for waxy surfaces) drop methods, where water drops were placed on a horizontal leaf surface. The detailed description of the method is given in Gorb and Gorb (2006). We used 1 μl droplets and circle/ellipse fitting for evaluation of apparent contact angles. Data are presented in the text as mean ± SD for *n* = 20 leaves of 10 individual plants for each species.

### Icing experiments in the Cryo-SEM

Small samples (1 cm × 1 cm) from the middle part of the leaves were cut out from living plants, immediately attached with their lower side to the metal holder using polyvinyl alcohol Tissue-Tek® OCTTM compound, and frozen in the vicinity of liquid nitrogen. Water vapor from the air generated icing, the intensity of which on the plant surface was dependent on the time of exposure to the liquid nitrogen. Usually, we stopped icing formation after 1–2 min and quickly transferred the sample in a vacuum to the cryo stage preparation chamber at − 140 °C. Frozen samples were not sputter-coated but directly studied in the frozen condition in the Cryo-SEM Hitachi S-4800 at 3 kV accelerating voltage and − 120 °C temperature.

After examination, samples were taken out of the microscope and held under room conditions for 3 – 5 min until the ice crystals started to thaw and then quickly frozen by submersion into the liquid nitrogen (1–2 min) and transferred in vacuum to the cryo stage preparation chamber at − 140 °C. Frozen samples were directly studied in the frozen condition in the Cryo-SEM (3 kV accelerating voltage, − 120 °C temperature). After examining for ice damage on the plant surfaces, samples were transferred back to the cryo stage preparation chamber at − 140 °C. Ice was sublimated (freezing-drying) by heating the samples up to − 90 °C and further studied in their frozen condition in the Cryo-SEM (3 kV accelerating voltage, − 120 °C temperature).

It is important to note that the Cryo-SEM experimental conditions are different from environmental conditions in many respects. The freezing temperature in the vicinity of the liquid nitrogen is difficult to control, and at some point of the experiment, we had to go to − 140 °C in order to keep the formed surface ice under the vacuum condition of SEM. Plants under natural conditions are never exposed to such low temperatures. In spite of these differences, this experiment clearly shows the interaction between the ice, formed from the water vapor, and cold plant surfaces. In the description of the results, we use the term *lightly iced* condition, when single scattered ice crystals up to a monolayer of ice crystals are situated on the surface, whereas *heavily iced* condition contains many layers of ice crystals.

## Results

### Micromorphology and wettability of upper leaf plant surfaces studied

**Fig. 4:**
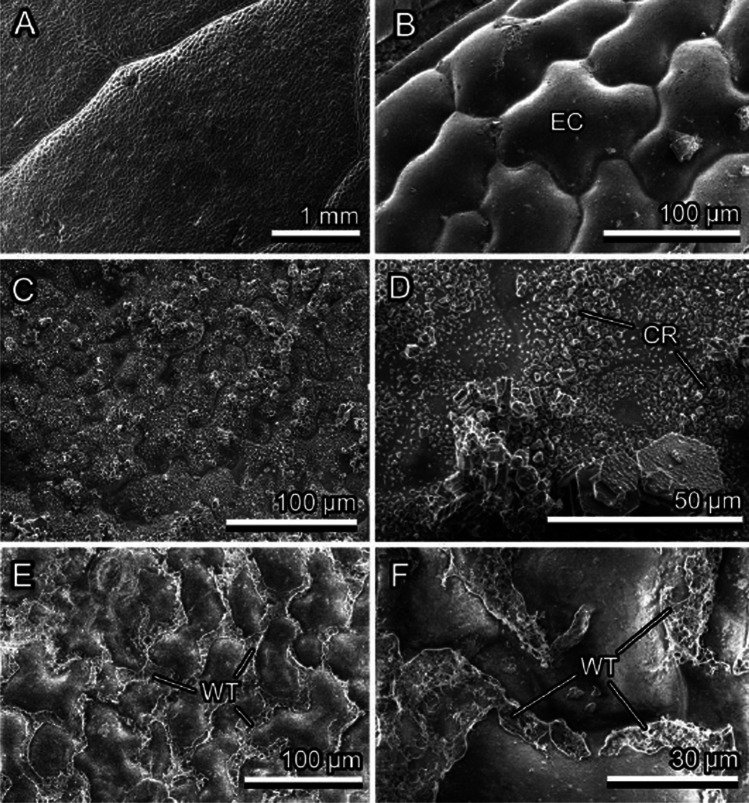
*Ficaria verna*, smooth upper surface of the leaf, Cryo-SEM experiment: native, non-iced surface (pattern of epidermal cells (EC) is visible) (**A**, **B**); lightly iced surface (clusters of ice crystals (CR) are mainly visible on the elevated tops of epidermal cells) (**C**, **D**); and iced surface after short thawing (re-iced water (WT) is visible in the depressions at the borders between epidermal cells) (**E**, **F**)

**Fig. 5:**
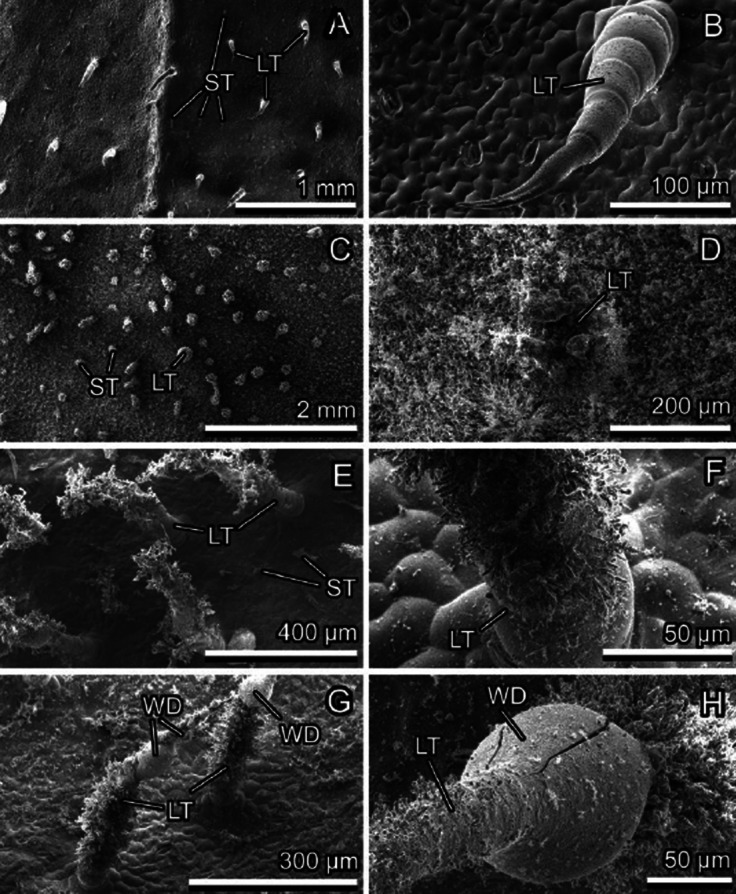
*Bellis perennis*, upper surface of the leaf, cryo-SEM experiment: native, non-iced surface with large (LT) and small (ST) trichomes (**A, B**); heavily iced surface (large clusters of ice crystals indicate positions of small and large trichomes) (**C**); heavily iced surface (the large trichome is visible in the center of the image) (**D**); light-iced surface (ice crystals are present only on the large multicellular trichomes) (**E**); basis of the large trichome covered with ice crystals (the plane surface of the leaf is almost uncontaminated with ice) (**F**); and iced large trichomes after short thawing (re-iced drops of water (WD) are visible) (**G, H**)

**Fig. 6:**
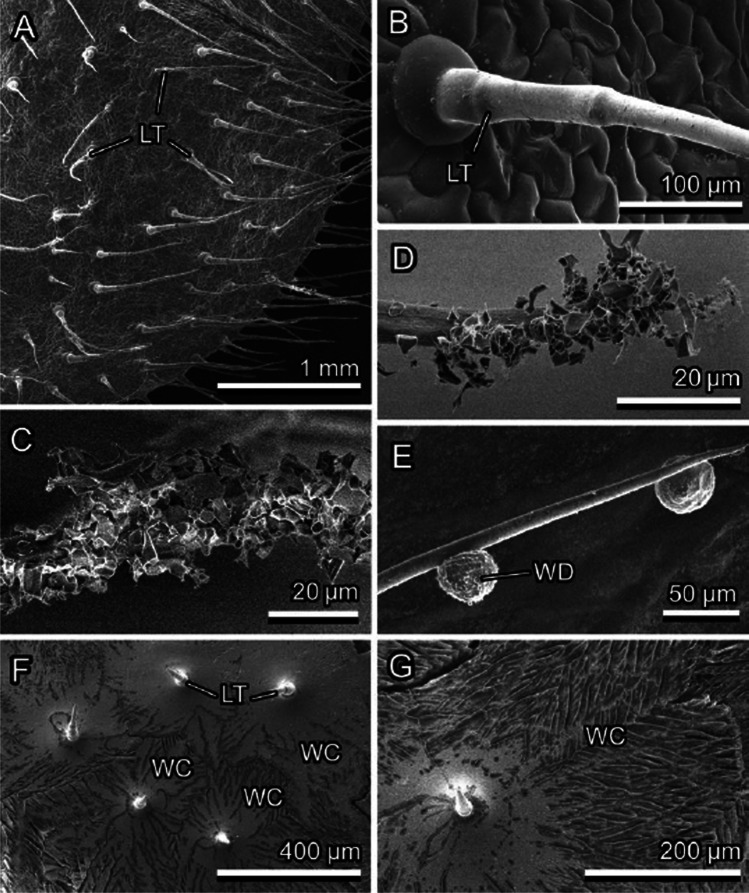
*Cerastium brachypetalum*, upper surface of the leaf, Cryo-SEM experiment: native, non-iced surface with large trichomes (LT) (**A, B**); lightly iced surface (clusters of ice crystals are visible on the tips of trichomes) (**C, D**); iced trichomes after short thawing (re-iced drops of water (WD) are visible) (**E**); and heavily iced leaf surface after short thawing (re-iced water (WC) builds clusters around bases of trichomes, which act as centers of crystallization) (**F, G**)

**Fig. 7:**
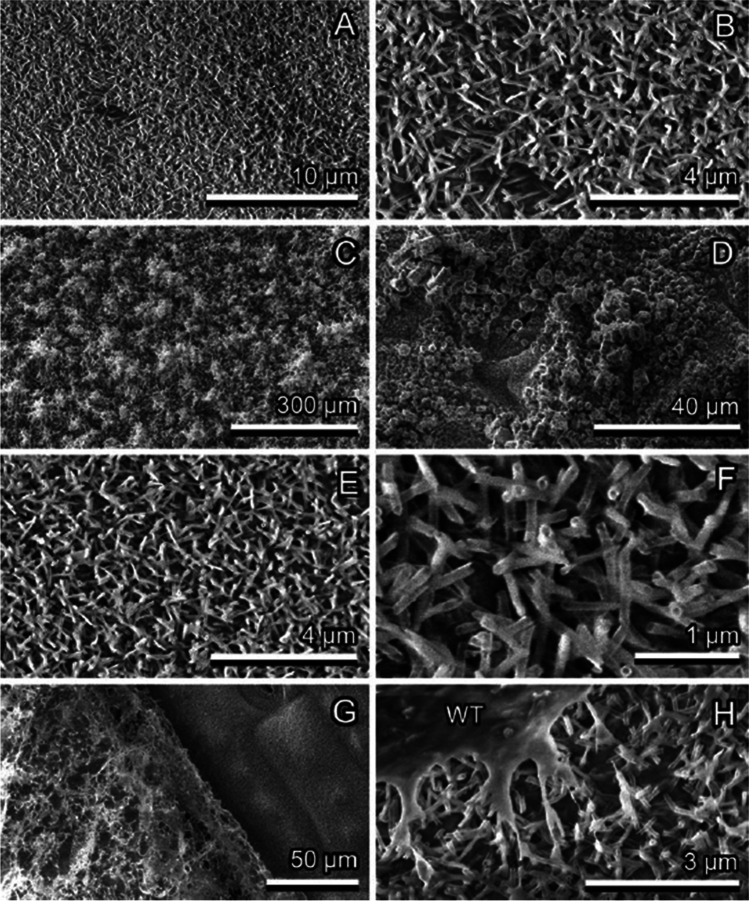
*Tulipa gesneriana*, wax-bearing upper surface of the leaf, Cryo-SEM experiment: native, non-iced surface (**A, B**); heavily iced surface **(C, D**); heavily iced surface after short thawing **(E, F**); and heavily iced surface after short thawing and re-freezing (re-iced water (WT) is visible) (**G, H**)

*P. laurocerasus* and *F. verna* have rather smooth upper leaf surfaces. *P. laurocerasus* bears a relatively thick and mechanically stable cuticle (together with the smooth epicuticular wax), and epidermal cell shapes are only hardly visible in SEM, whereas *F. verna* (Fig. 4A, B) with its thin cuticle (together with the smooth epicuticular wax) bears a distinct surface pattern of epidermal cells well recognizable in SEM. *B. perennis* surface is covered with long multicellular, uniseriate trichomes. The trichomes have dart-like shapes, and their diameter becomes narrower toward the sharp tip (Fig. 5A, B). Trichome cell side walls are slightly convex. Additionally, very small trichomes are scattered between large ones on the surface. *C. brachypetalum* surface is covered only with long multicellular, uniseriate, dart-shaped trichomes, having a sharp tip and a socket at the base (Fig. 6A, B). Trichome cell side walls are slightly concave. *N. pseudonarcissus* and *T. gesneriana* leaves are rather uniformly covered by 3D epicuticular waxes (Fig. 7A, B). The wax projections of *T. gesneriana* represent slender nanoscopical tubules (length: 0.68 ± 1.17 μm, outer diameter: 0.11 ± 0.01 μm, inner diameter: 0.06 ± 0.01 μm), whereas those of *N. pseudonarcissus* are nanoscopical irregular platelets (length: 0.93 ± 0.32 μm, width: 0.46 ± 0.05 μm, thickness: 0.07 ± 0.02 μm).

Contact angles of water were 97 ± 5° on *P. laurocerasus*, 77 ± 13° on *F. verna*, 88 ± 22° on *B. perennis*, 93 ± 9° on *C. brachypetalum*, 152 ± 7° on *N. pseudonarcissus*, and 157 ± 8° on *T. gesneriana.*

### Ice formation on smooth plant surfaces

Ice on smooth plant surfaces started with regularly distributed nucleation sites (Figs. 3A, 4C, D, and 9A, B), which, with an increased amount of frozen water vapor, built hexagon-shaped crystals (Fig. 4D). The initial nucleation sites formed rather large contact areas with epidermal cell surfaces. These pioneer crystals served as further nucleation sites and then developed further into macroscopical needles (Fig. 3B). Similar to the observation on the leaf under natural conditions, the formation of macroscopical ice structures was strongly dependent on the environmental conditions.

After thawing, fluid water filled the depressions between elevated middle parts of epidermal cells (Fig. 4E, F). Water has a certain affinity to these leaf surfaces, especially strong in *F. verna*; that is why the contact area between fluid water and epidermal cell surfaces became very large.

### Ice formation on plant surfaces covered with trichomes

Ice formation on hairy plant surfaces always started with nucleation on long trichomes (Figs. 3C, D, 5E-H, 6C, D, and 9E, F). With an increased amount of frozen water vapor, the nucleation was further observed on small, densely distributed trichomes and only afterwards on the surface of epidermal cells (Fig. 5C, D). Similar behavior was also observed in nature on many other plant species, for example *Campanula portenschlagiana* and *Fragaria ananassa* (Fig. 1G, H).

After thawing, fluid water usually remained on the trichome surface and after water freezing from this fluid condition, the frozen water droplets remained attached to the trichomes and did not contact the surface of epidermal cells (Figs. 5G, H, and 6E). Due to this freezing behavior, epidermal cells have less of a chance to be frozen down quickly and be damaged by the water layer frozen on the surface (Fig. 9G and H). However, if the amount of water is so high that it can no longer be kept on the trichome surface and if the second level of smaller trichomes is absent (as in *C. brachypetalum*), water can penetrate the boundary layer and form ice crusts directly on the surface of epidermal cells. In this case, trichomes usually become sites of ice nucleation and the ice crystal orientation is clearly dependent on the arrangement of trichomes on the surface (Fig. 6F, G).

### Ice formation on plant surfaces covered with epicuticular wax projections

**Fig. 8:**
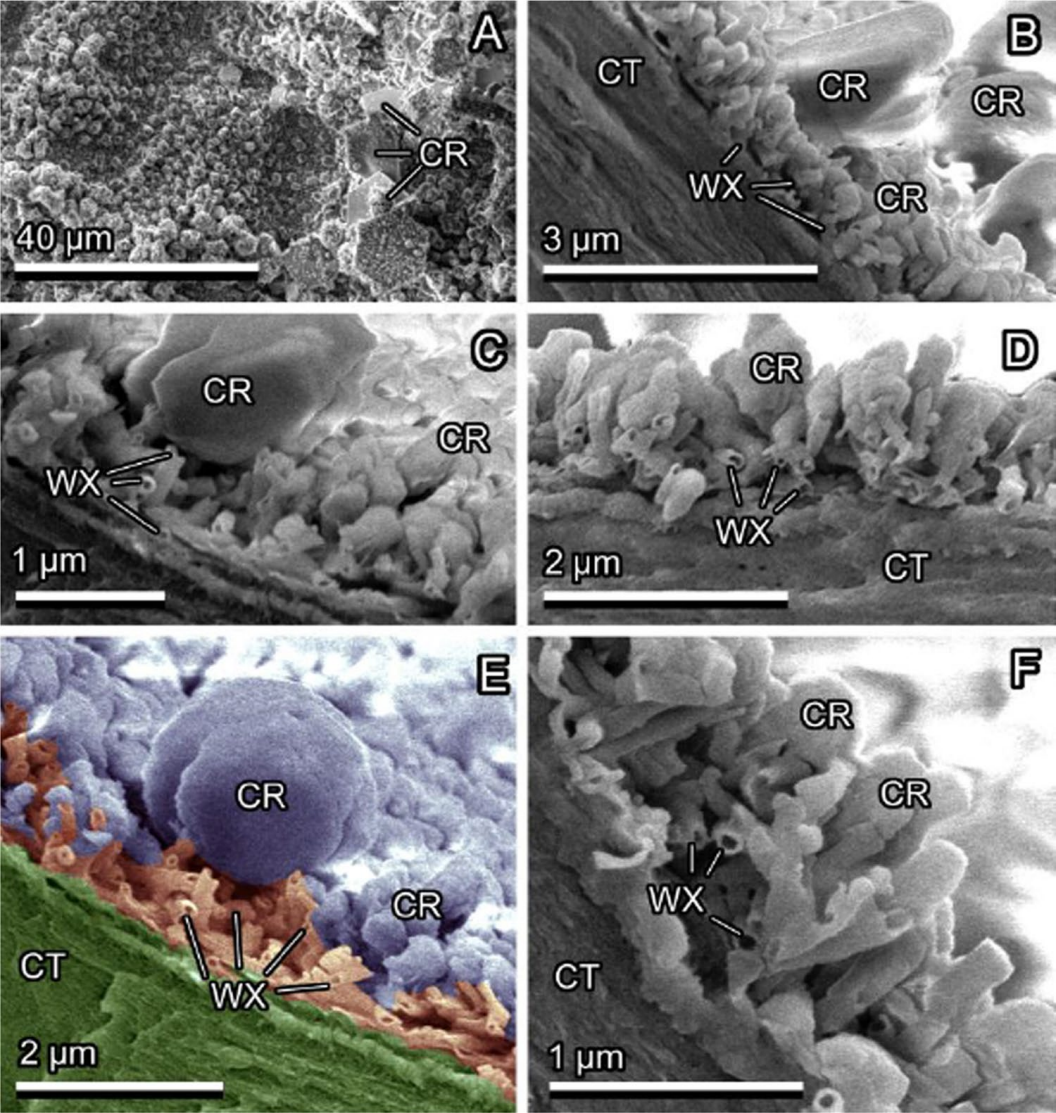
*Tulipa gesneriana*, wax-bearing upper surface of the leaf, Cryo-SEM experiment: heavily iced surface showing ice crystals (CR) (**A**); cryo fractured heavily iced surface (ice crystals are visible on and between single wax projections (WX)) (**B–F**). In (**E**), ice crystals are colored lilac, wax projections are colored brown, and the cell wall (CT) is colored green

**Fig. 9:**
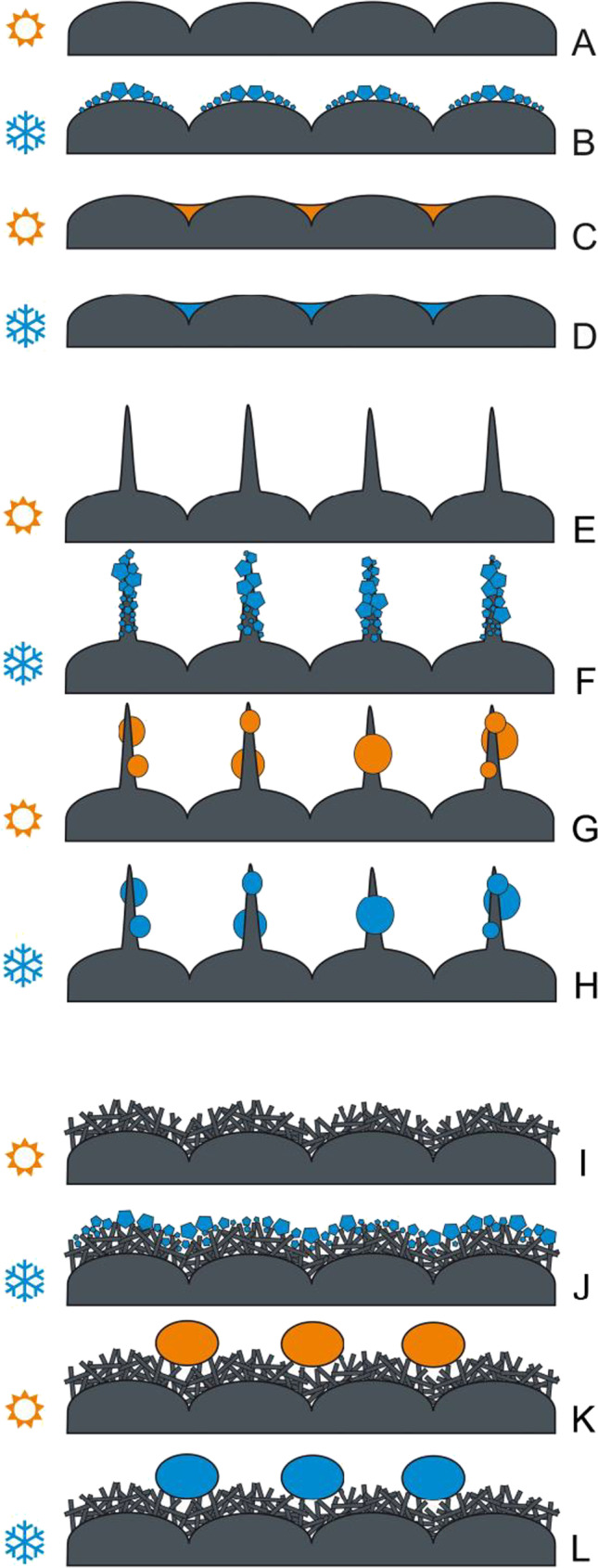
Diagram of the surface icing (**B, F, J**), thawing (**C, G, K**) and re-icing (**D, H, L**) on a smooth (without either trichomes or 3D wax) plant surface (**A**) and plant surfaces covered by either trichomes (**E**) or 3D wax coverage (**I**). Orange colored labels and structures indicate warm conditions above 0 °C. Blue colored labels and structures indicate cold conditions below 0 °C

Ice forming on plant surfaces covered with 3D epicuticular wax at first, at low magnification of the light microscope or Cryo-SEM, did not differ from that on smooth surfaces (see “Ice formation on smooth plant surfaces”). If one compares binocular microscope images Fig. 3B (*F. verna*, smooth) with Fig. 3E, F (*T. gesneriana*, 3D wax), or Cryo-SEM images Fig. 4C (*F. verna*, smooth) with Fig. 7C and D (*T. gesneriana*, 3D wax), it is hard to recognize any difference. It seems that ice formation on plant surfaces covered with wax projections started at more or less regularly distributed nucleation sites, which, with an increased amount of frozen water vapor, built larger hexagonal ice crystals (Figs. 7C, D, 8A, and 9I, J). Later, these crystals serving as further nucleation sites developed into macroscopical needles (Fig. 1E, F). The initial nucleation sites formed rather large areas, but they were not in contact with the epidermal cells’ surface, because they were initiated on the surface of wax projections or in nanodepressions between them (Fig. 8B-F). That is why, in spite of the large apparent contact area to the surface, the real contact area of ice crystals is limited to a few sites at the nanoscale on or between wax projections (Fig. 8E).

After thawing, water drops rolled off the surface, even at very small angles (2–3°). This behavior, due to the surface superhydrophobicity, immediately cleaned the surface of water that could be potentially re-frozen and in frozen conditions represents potential danger of further damage to the plant surface (Fig. 9K, L). Due to fluid water escaping from these surfaces, it was really difficult in our experiment to generate images of water fluid drops frozen in contact (Fig. 7G, H). In this case, water adhered to the sites containing defects in the 3D wax layer.

Due to the fact that ice crystals were often deposited between single wax projections, we expected strong damage in the 3D wax layer on iced surfaces. However, the wax layer remained absolutely intact after ice thawing and water escaping from the surface (Fig. 7E, F).

## Discussion

### Effects of surface wettability and roughness

It is well known that ice can be formed on surfaces in general and on outer surfaces of plants in particular. Depending on environmental conditions, this ice can be formed quickly and in large amounts. The extracellular surface structures as well as the outermost cell walls of epidermis can stop the nucleation of ice crystals from moving inside cells, but depending on the contact area between ice and plant surface, the freezing rate within the cell’s interior might be potentially different. Additionally, some smooth plant cuticles are rather thin and vulnerable to potential mechanical damages by ice. That is why it was plausible to assume that the structure and chemistry of plant surfaces may influence plant resistance against damages caused by freezing.

Plant surfaces vary greatly in their texture (Gorb and Gorb 2013, 2017) and wettability (Neinhuis and Barthlott 1997), which may lead to different degrees of ice nucleation on the plant. Our experiments clearly show that icing, thawing, and re-freezing on plant surfaces strongly depend on the surface structure and property. Surfaces that are smooth at micro- and nanoscales are more susceptible to ‘icing,’ because ice crystals are directly formed at the cuticle surface (Fig. 9A, B) building a large contact area to the epidermis and ice crystals are situated very close to the cell’s interior water. Since surfaces of the studied plant species with smooth leaves are not strongly hydrophobic, after thawing, water is readily spread over the surface and, when freezing in this condition, ice might quickly cool down the inner content of epidermal cells (Fig. 9C, D). Additionally, due to the volume increase during freezing, such ice crusts might potentially generate some lateral forces on the cells in contact and cause mechanical damage. However, the situation strongly depends on the external temperatures. When ice is formed on the surface, it can provide some thermal insulation of the leaf and even prevent further freezing of the cell interior. This effect is used by fruit growers spraying water on their trees to protect them from frost damage.

Non-smooth plant surfaces show different ‘icing’ behaviors and freezing behaviors of water after thawing. In trichome-covered plant surfaces, cooling down of the epidermal cells is postponed due to the initial ice formation on trichomes. It seems that the trichome coverage keeps the boundary layer of air rather dry after initial ‘icing’ of trichomes and leads to a delay of the water vapor access from the outside to the boundary layer close to epidermal cells (Fig. 9E, F). Since trichomes of the plant species studied are rather hydrophilic, after thawing and re-freezing, ice remains on the trichome surface and the boundary air layer remains intact further protecting plants from ‘icing’ and freezing their cell interior (Fig. 9G, H).

Surfaces covered by 3D wax projections are highly hydrophobic or superhydrophobic (Neinhuis and Barthlott 1997; Barthlott et al. 2016), which aids in keeping them dry during/after the rain or after the morning dew. This automatically prevents the freezing of water at a large contact area of the leaf. Additionally, ice crystals coming from the vapor will be formed at some distance from the cuticle and epidermis. This prevents rapid freezing of the cell interior (Fig. 9I, J). Epicuticular waxes have been previously implicated in frost resistance of leaves in *Triticum aestivum* and *Eucalypthus urnigera* (Single and Marcellos 1974; Thomas and Barber 1974). It has also been shown that smooth plant surfaces covered by artificial hydrophobic particles limit spreading of ice crystal formation and delay plant freezing (Fuller et al. 2003). Subsequent examination of the freezing of leaves revealed that the hydrophobic particle film delayed the entry of ice from a frozen water droplet. In the present paper, we demonstrate that the mechanism of this delay is based on the air pocket buffer between ice particles and the plant cuticle. Furthermore, this study shows that plants naturally possessing superhydrophobic surfaces are, to a certain extent, protected from rapid freezing from an icy surface.

### Ecological aspects

Freezing resistance is not constant for a given plant, but varies, being driven by seasonality and short-term acclimation. Some alpine taxa have been found to perform leaf super-cooling, which is a phenomenon of retaining water in a gel-like meta stable state below the freezing point to avoid ice nucleation (Körner 2016). Freezing avoidance is the second important evolutionary adaptation to life under seasonally cold conditions, which is given by the species-specific phenological plant adaptations. Phenological timing in Spring leads to the avoidance of the exposure of vulnerable tissues to freezing (Sakai and Larcher 1987). Avoiding freezing and freezing tolerance are, in many plants, coupled mechanisms and both are essential for the adaptation to cold temperatures. Phenological, biochemical, and physiological adaptations of plants to subzero temperatures are rather well studied in the literature. However, much less is known about morphological and ultrastructural adaptations at the level of plant surfaces.

A great number of surfaces of different plant organs and in different plant species are covered with trichomes, which are usually hair-like protuberances extending from the epidermis of aerial plant tissues. The diversity of the structural types of trichomes is immense. They vary in indumentums (coverage of fine hairs), orientation, bases, cellular arrangement, branching, shape, etc. (see review Gorb and Gorb (2013)). Trichomes occur regularly or irregularly, sparsely or densely dispersed on the surface. They are often aligned in one preferred direction, although some have a perpendicular orientation to the underlying surface. In a number of cases, trichomes form a compact, felt-like coverage on the surface. From the functional point of view, trichomes have been considered to be adaptive to improving plant climbing abilities, increasing the hydrophobicity of the surface, decreasing wind velocity on the plant surface, and preventing water loss (Jeffree 1986). They can diminish leaf absorptance to solar radiation (Ehleringer et al. 1976, 1981) and reduce leaf temperature (Housman et al. 2002). Trichomes also contribute to defense mechanisms against herbivorous insects (Voigt et al. 2007; Gorb and Gorb 2013). The present paper clearly demonstrates the functional role of trichomes in preventing and delaying ice formation by being the nucleation points for the formation of ice from vapor and in protecting the plant surface from overcooling, when fluid water freezes in contact with the leaf surface. Many early spring ephemeroids, such as representatives of the genera *Pulmonaria*, *Pulsatilla*, and *Hepatica*, are heavily covered by long trichomes, which might be an adaptation to occasional subzero temperatures. It was previously shown that leaves of subalpine and alpine plants are highly nonwettable and the adaxial pubescence occurs more frequently (Aryal and Neuner 2010). Such an altitudinal wettability gradient in plant leaf properties is explained by the fact that lower wettability turns water droplets into a spherical form allowing them to drip off much more easily. Structural features of the surface for low wettability are developed in cold environments and open sites with frequent dew formation as it appears to be an important functional trait to prevent a number of negative effects adhering surface water may have in such an environment (Aryal and Neuner 2010). Unfortunately, data on leaf wettability depending on latitudinal gradient are missing in the literature.

A comprehensive survey of the construction principles and occurrences of superhydrophobic surfaces in plants, animals, and other organisms based on literature data and SEM examinations of 20 000 species (Barthlott et al. 2016) discussed such functions of these surfaces as self-cleaning (lotus effect), air-retaining, and fluid drag reducing (*Salvinia* effect), but no evidence for their role in reduction of the freezing damage of plant surfaces was demonstrated. Our study shows two important effects that might reduce plant cell freezing rate: (1) the presence of air pockets between wax projections that protect from direct contact between ice crystals and cuticle (Fig. 9I, J) and (2) elimination of fluid water after thawing and preventing further re-freezing on the surface (Fig. 9K, L). The first effect is demonstrated here for the first time. The superhydrophobic particle coverage including natural 3D wax projections might serve as frost protection agents that naturally occur on the surface or might be applied to plants prior to a freezing event (Fuller et al. 2003). Many crops such as wheat or rapeseed that over-winter in the temperate zones and are exposed to rather low winter temperatures posses such a protection. Many early spring ephemeroids, such as species from the genera *Narcissus*, *Corydalis*, *Galanthus*, *Dicentra*, and *Tulipa*, are heavily covered by 3D wax projections, which might be additional adaptations against freezing. This fact might inspire breeding of the cultivars with superhydrophobic wax coverage to increase their resistance against subzero temperatures.

### Biomimetic aspects

Ice on many technical surfaces can lead to some catastrophic events and that is why engineering literature of recent years is full of developments of so-called anti-icing surfaces. The standard strategy in engineering is de-icing of already iced surfaces using a mechanical approach, electro-impulses, and chemical methods. Recent developments of passive anti-icing surfaces are rather impressive (see review by Li and Guo (2018)). Researchers considered numerous physical phenomena to reach this goal: the kinetics of ice nucleation, ice accretion on solid surfaces, and heat transformation during ice formation. Generally, there are two main strategies in these developments. The first one is anti-icing, which is based on (a) the reduction of ice adhesion to the surface, (b) decrease of nucleation temperature, and (c) increasing of freezing time. The second strategy is ice-phobicity, which is basically liquid removal before it starts to freeze. The latter strategy is nothing other than the well-known superhydrophobicity (Neinhuis and Barthlott 1997; Barthlott et al. 2016) and contains three ways to remove fluid water: (a) by drop rebound, (b) by shear flow shedding, and (c) by drop rolling-off.

Some researchers claim employment of passive anti-icing and ice-phobic surfaces inspired by animals and plants (Li and Guo 2018), but in our opinion, the mechanisms and functional principles of these phenomena in biological surfaces have not been previously studied (except for a very intensely studied phenomenon of superhydrophobicity). Therefore, it remains difficult to use data from biology for successful biologically-inspired technical solutions for anti-icing. In addition to a wide usage of superhydrophobic surfaces, biologically inspired anti-icing surfaces may be created via the direct immobilization of anti-freeze proteins on the surface (Gwak et al. 2015) or by anti-freeze-secreting anti-icing coatings (Sun et al. 2015; Wang and Guo 2019). The present study provides an additional interesting mechanism of using hairy surfaces (especially multilevel hairy surfaces) to increase freezing time of the surface by shifting ice nucleation from the surface to hairs and keeping a boundary air layer between the hairs in a water–vapor-reduced condition. Also, using superhydrophobic surfaces in combination with high air flow can remove already formed ice particles, due to their low real contact area with the nanoscale surface (as observed here in *T. gesneriana*), and therefore, their low adhesion/friction on the surface (Liu et al. 2019; Xu et al. 2020).

## Conclusions

In the present study, we aimed at visualization of icing on plant surfaces having varying micro- and nanostructure by studying six plant species with rather smooth surfaces and those covered with trichomes or 3D epicuticular wax. Icing on smooth plant surfaces generated large contact areas between ice and epidermal cells with a strong danger of further freezing of the cells. After thawing and re-freezing, contact area between fluid water and epidermal cell surface became even larger. Hairy plant surfaces delayed ice crystal formation on the cell surface by keeping the boundary layer of air rather dry after initial icing of trichomes and by preventing the access of water vapor from the outside to the boundary layer near the surface of epidermal cells. After thawing, fluid water usually remained on the trichome surface and after re-freezing, the frozen water droplets remained on the trichomes and did not contact the surface of epidermal cells. Icing on plant surfaces covered with 3D wax projections started from the nucleation sites on the surface of wax projections or in nanodepressions between them. That is why the real contact area of ice crystals was limited to a few sites at the nanoscale on or between wax projections. After thawing, water drops rolled off the surface immediately cleaning the surface of water that could be potentially re-frozen. The 3D wax layer remained intact after ice thawing. Our Cryo-SEM experiments clearly demonstrated that trichome coverage (especially with several distinct layers) and 3D wax coverage can be recognized as anti-icing strategies of plants.

## Data Availability

The data generated during and analyzed during this study are available from the corresponding author upon reasonable request.

## Code availability

Code was generated from Software SigmaScan Pro Version 5.0.0 (SPSS Inc., Chicago, USA).
